# Vertically-Aligned Functionalized Silicon Micropillars for 3D Culture of Human Pluripotent Stem Cell-Derived Cortical Progenitors

**DOI:** 10.3390/cells9010088

**Published:** 2019-12-30

**Authors:** Alessandro Cutarelli, Simone Ghio, Jacopo Zasso, Alessandra Speccher, Giorgina Scarduelli, Michela Roccuzzo, Michele Crivellari, Nicola Maria Pugno, Simona Casarosa, Maurizio Boscardin, Luciano Conti

**Affiliations:** 1 Laboratory of Stem Cell Biology, Department of Cellular, Computational and Integrative Biology-CIBIO, University of Trento, 38123 Trento, Italy; alessandro.cutarelli@unitn.it (A.C.); Jacopo.Zasso@unitn.it (J.Z.); 2 Fondazione Bruno Kessler-Center for Material and Microsystem, 38123 Trento, Italy; simoneghio1@gmail.com (S.G.); crivella@fbk.eu (M.C.); 3 Laboratory of Neural Development and Regeneration, Department of Cellular, Computational and Integrative Biology-CIBIO, University of Trento, 38123 Trento, Italy; a.speccher.1@unitn.it (A.S.); simona.casarosa@unitn.it (S.C.); 4 Advanced Imaging Facility, Department of Cellular, Computational and Integrative Biology-CIBIO, University of Trento, 38123 Trento, Italy; Giorgina.Scarduelli@unitn.it (G.S.); michela.roccuzzo@unitn.it (M.R.); 5 Laboratory of Bio-Inspired and Graphene Nanomechanics, Department of Civil, Environmental and Mechanical Engineering, University of Trento, 38123 Trento, Italy; nicola.pugno@unitn.it; 6 School of Engineering and Materials Science, Queen Mary University of London, London E1 4NS, UK; 7 Ket-Lab, Edoardo Amaldi Foundation, via del Politecnico snc, I-00133 Roma, Italy

**Keywords:** human cortical progenitors, 3D culture, silicon pillars, cell growth, hiPSC-derived neural progenitors, cerebral cortex

## Abstract

Silicon is a promising material for tissue engineering since it allows to produce micropatterned scaffolding structures resembling biological tissues. Using specific fabrication methods, it is possible to build aligned 3D network-like structures. In the present study, we exploited vertically-aligned silicon micropillar arrays as culture systems for human iPSC-derived cortical progenitors. In particular, our aim was to mimic the radially-oriented cortical radial glia fibres that during embryonic development play key roles in controlling the expansion, radial migration and differentiation of cortical progenitors, which are, in turn, pivotal to the establishment of the correct multilayered cerebral cortex structure. Here we show that silicon vertical micropillar arrays efficiently promote expansion and stemness preservation of human cortical progenitors when compared to standard monolayer growth conditions. Furthermore, the vertically-oriented micropillars allow the radial migration distinctive of cortical progenitors in vivo. These results indicate that vertical silicon micropillar arrays can offer an optimal system for human cortical progenitors’ growth and migration. Furthermore, similar structures present an attractive platform for cortical tissue engineering.

## 1. Introduction

In biology, developing tissues’ microarchitecture is fundamental to allow correct cell differentiation and organization into appropriate structures that relate to specific physiological functions [1,2]. Standard in vitro cell culture models are mainly set in reductionist monolayer settings, conditions that intrinsically lack any structural architecture. This condition often represents a poor proxy to extrapolate cell growth in vivo, thus substantially affecting cell performance and biological assays outcomes [3,4]. Indeed, with respect to whole tissues, monolayer cultured cells are usually more responsive to toxic or therapeutic agents [5,6]. Additionally, cell culture on rigid surfaces can enhance cell proliferation, but might impact cell differentiation due to the partial cell interactions [7].

In this view, more appropriate three-dimensional (3D) cell culture environments can allow more physiological cell-to-cell contact, assisting cell growth and allowing better modelling of developmental processes [8,9,10]. A 3D environment also facilitates cells to organize into tissue-like structures that better mimic the in vivo function of cells, thus enhancing physiological relevance and predictive accuracy [11,12,13,14]. In the last few years, there has been a gradual development and adoption of technologies that enable tissue-like 3D cultures. Both scaffold-free organoid-based technologies and natural or synthetic scaffold-based culture systems have been developed [7]. In particular, since different tissue types show definite assemblies associated with their functional organization, scaffold-based methods allow assisted mimicking of complex tissue geometrical topographies, such as cerebral cortex, thus facilitating effective biofabrication of in vitro 3D tissue-like models [15,16,17]. With this purpose, silicon-based micro-fabricated culture substrates with well-defined continuous and discontinuous topographies, including the development of surfaces patterned with grooves, nanopillars or nanowires for the study of neural guidance and polarity, have been extensively developed in order to create scaffolds for a variety of applications [18,19,20,21,22,23,24].

In the present study, we exploited vertically-aligned silicon micropillar arrays to reproduce the developing cerebral cortex 3D architecture, where effective control over neural columns width, resembling the mammalian neocortex, is required for a large spectrum of applications. In the developing cerebral cortex, cortical progenitors are oriented from the ventricular to the pial surface with an apical-basal polarity. They divide to form radial glia and neuroblasts, the latter can migrate using the apico-basal oriented radial glia fascicles of the subventricular zone (SVZ) as a scaffold, thus forming the different cortical layers [25,26]. Here we focused on mimicking the radially-oriented cortical radial glia fibers, as they are the key players in controlling the expansion, radial migration and differentiation of cortical progenitors, thus allowing for the establishment of the correct multilayered cerebral cortex structure. We show that the scaffold material and structures are compatible with human cortical progenitors’ maintenance. Immunofluorescence imaging analysis and RT-PCR results reveal that silicon vertical micropillar arrays efficiently promote the expansion and stemness preservation of human cortical progenitors, when compared to monolayer growth conditions. Furthermore, the precise orientation of the micropillars allows the radial migration, movement that is distinctive of cortical progenitors in vivo. These results indicate that vertically-aligned silicon micropillar arrays can offer an optimal system for human cortical progenitor growth and migration, and a potentially interesting platform for cortical tissue engineering.

## 2. Materials and Methods

### 2.1. Vertically-Aligned Silicon Micropillar Array Fabrication

Silicon slides containing vertically-aligned micropillar arrays have been realized on silicon surface through a CMOS like process at the Micro-Nano Facility of Fondazione Bruno Kessler (FBK). The silicon slides are composed of a central zone containing a matrix of vertically-aligned micropillars (height: 250–600 µm). Slides with different micropillar diameters have been obtained, with a diameter of 10 or 15 µm that leads to an aspect ratio that varies from 1:25 to 1:17. These significant proportions are particularly hard to achieve for pillar-like structures. The classical MEMS devices usually have aspect ratio of 1:12, for this reason the Bosch process used to realize the surfaces has been tuned in order to be able to reach higher aspect ratio.

The slide surface used for cellular seeding and culturing is 14 mm long and 5 mm wide these sizes were specifically designed to allow each slide to be accommodated in a well of a 12-well cell culture plate. The design foresees a range of distances between the micropillars, from 20 to 50 µm to evaluate the advantages of smaller and larger spaces between the micropillars for cellular deposition and growth. To realize the structures, the silicon wafer was first oxidized, patterned through soft lithography, then the silicon dioxide (SiO_2_) was patterned through dry etching, and finally a deep reactive ion etching (DRIE) process was used to realize the micropillar-like structures. The DRIE process consists of a two-step process: first an isotropic plasma etch, second the deposition of a passivation layer that protects the lateral part of the structure. This two-step process is called Bosch process and it is repeated several times to realize nearly vertical wall. The time lapse of each of the two steps defines the roughness of the vertical wall as the process feed rate while the maximal depth that can be reached is defined by the power of the radio frequency bias that accelerates the ions toward the surface.

Since the fabrication process does not damage the mask, the same stamp can be employed for many subsequent cycles in a very reproducible manner.

During slides production, the micropillars on the open edge of the structures are systematically damaged during the DRIE process. This effect is connected with the presence of a blank space between slides, this void space increments the effective power of the ions impacting on the surfaces and then destroy or modify the shape of the first micropillars row. These defects have been eliminated in the cutting step. The cutting diamond disk was placed over the border, in order to cut away the defected micropillars and leaving a sharp edge.

### 2.2. Cell Cultures

Human cortical progenitors used in this study were differentiated from a commercial control hiPSC line (Gibco, Thermo Fisher Scientific, Monza, Italy) as previously described [27,28]. Briefly, hiPSCs commitment to a neural lineage and subsequently to the dorsal telencephalic lineage was performed by using N2B27 supplemented with human recombinant Noggin (500 ng/mL, Peprotech, London, UK), SB431542 (20 mM, Santa Cruz Biotechnologies, Heidelberg, Germany) and Fibroblast Growth Factor-2 (4 ng/mL, Peprotech). Cells were then detached and seeded on poly-ornithine and laminin-coated plastic dishes (Sigma-Aldrich, Milan, Italy) in medium supplemented with 10 μM Rock inhibitor Y27632 (Sigma-Aldrich). At day 10, neural rosettes containing cortical progenitor cells were manually collected and plated on poly-ornithine/laminin-treated culture dishes in N2B27 medium containing epidermal growth factor (10 ng/mL), fibroblast growth factor-2 (10 ng/mL) and brain-derived neurothrophic factor (20 ng/mL). Confluent cultures were passed as small multicellular clumps at a ratio of 1:3 using trypsin and amplified until passages 8–10. Cryopreserved stocks of human cortical progenitors were prepared from confluent cultures by trypsinization and resuspension in freezing medium (10% DMSO and 90% foetal calf serum).

Neuronal differentiation of hCPs was induced as previously described. Briefly, hCPs were plated at high density (10^5^ cells per cm^2^) on laminin-treated silicon vertical micropillar arrays. Cells were allowed to grow for four days and then switched in N2B27 medium without growth factors and cultured for 35 days. The medium was changed every four days.

To visualize the cells for fluorescent imaging analyses and time-lapse experiments, hiPSC-derived cortical progenitors were transduced with a lentiviral vector carrying eGFP cDNA under Cytomegalovirus (CMV) promoter and containing a puromycin selection cassette [29].

Procedure for generation and expansion of mouse NS cells (i.e., radial glia-like neural progenitors) were previously described [30,31,32]. In this study we used the LC1 NS cell line grown in standard conditions in expansion medium composed of Euromed-N medium (Euroclone) supplemented with 1% N2 (Thermo Fisher Scientific) and 20 ng/mL human recombinant epidermal growth factor (20 ng/mL) and fibroblast growth factor-2 (20 ng/mL).

Human cortical progenitors and mouse NS cells were seeded on silicon slides pre-coated with mouse laminin (Thermo Fisher Scientific).

### 2.3. Scanning Electron Microscopy (SEM)

Cultures were fixed for 30 min at 4 °C in 25% Glutaraldehyde and 0.1 M cacodylic acid in distilled water (pH 7.2). Then, samples were washed three times with 0.1 M cacodylic acid and dehydrated by the exposure to 50%, 70%, 90% and (2×) 100% *v/v* ethanol concentration, 10 min each. After being air-dried under an air flow, samples were gold coated by evaporation of a thin gold layer on top of the sample surface (thickness 6 nm, 1.5 nm Cr adhesion layer). Silicon micropillar-based devices deprived of cells did not require any treatment prior to SEM image acquisition. SEM micrographs were acquired by using a TESCAN VEGA III scanning electron microscope (Tescan Analytics, Fuveau, France) (operating voltage 4 kV, working distance 18 mm, stage tilting angle 45°).

### 2.4. Cell Growth/Viability Assay

Analysis of cell growth/viability of all cell types employed in this work (hCPs and mouse NS cells) was performed by MTT assay (Sigma-Aldrich). Briefly, MTT powder was dissolved into culture medium at a final concentration of 1.5 mg/mL. For culture incubation with MTT solution, cell medium was removed, cultures rinsed twice with PBS (Thermo Fisher Scientific) and incubated with MTT solution for 1h at 37 °C. Following incubation, MTT solution was removed, cells were air-dried and violet MTT precipitates dissolved with isopropanol. The absorbance was read at 570 nm wavelength with a microplate reader (Tecan Infinite M200PRO, Tecan Italia, Milan, Italy).

### 2.5. Immunocytochemistry

To process samples for immunofluorescence analyses, cultures were fixed in 4% paraformaldehyde for 30 min at room temperature (RT), permeabilized in PBS containing 0.5% Triton X-100 for 15 min at RT and then blocked in blocking solution (PBS containing 0.3% Triton X-100 and 5% FCS) for 1 h at RT. Samples were next incubated overnight at 4 °C with primary antibody diluted in antibody solution (PBS containing 0.2% Triton X-100 and 2% FCS), then washed three times with PBS and incubated for 2 h at RT with secondary antibodies. Samples were then counterstained with 1 μg/mL Hoechst 33,258 (Thermo Fisher Scientific) and further rinsed with PBS before proceeding with visualization. Fluorescent signals and Z-Stack of eGFP^+ve^ human cortical progenitors (12 slices of 7.7 μm each, shown at 7 fps) were detected using a Leica DMi8 microscope equipped with an Andor Zyla 4.2 PLUS, monochromatic, sCMOS sensor, 4.2 megapixel camera. Acquired images were processed with the open-source Fiji software (v2.0.0, open source under the GNU General Public License, Madison, WI, USA) [33].

Antibodies used in this study: primary mouse monoclonal anti-NESTIN antibody (R&D Systems, Minneapolis, MN, USA, 1:300), primary mouse monoclonal β3-TUBULIN antibody (Promega, Milan, Italy, 1:1000), primary rabbit polyclonal anti-SOX2 antibody (Millipore, Milan, Italy, 1:200), primary rabbit polyclonal anti-MAP2 antibody (Santa Cruz, Heidelberg, Germany, 1:200), primary mouse monoclonal anti-TBR2 antibody (ABCAM, Cambridge, UK, 1:500), primary mouse monoclonal anti CUX1 (ABCAM, 1:200), AlexaFluor-488 or -568 conjugated secondary antibodies (Thermo Fisher Scientific, 1:500).

### 2.6. Time Lapse Analysis

Time-lapse movies of live GFP-expressing cells migrating along micropillars were acquired with a Zeiss Axio Observer Z1 inverted microscope equipped with the Apotome 2 module for structured illumination and a 2.83 Megapixel AxioCam 503 mono D (all from Zeiss Italia, Castiglione Olona, Italy). Time-lapses were acquired as z-stacks (10 μm z-step) using a plan-apochromatic 10×/0.3 objective, with a frame interval of 30 min for 12.5 h. The movies shown are maximum intensity projections. Optimal focus selection was performed by manual extraction of each focus z-slices from original z-stack time-lapses to select the best focused z position for each time point, then adjusted for brightness and contrast and saved as 7 fps AVI files using Fiji software [33].

### 2.7. RNA Isolation and Quantitative RT-PCR (qRT-PCR)

Total RNA was isolated by using TRIzol Reagent (Thermo Fisher Scientific) following the manufacturer’s protocol, then retro-transcribed with iScript cDNA Synthesis Kit (BioRad, Segrate, Italy). cDNA was used to verify the expression of specific target genes by qRT-PCR (quantitative RT PCR), using the SsoAdvanced Universal SYBR Green Supermix Kit. Specific primers sets were used (RT-qPCR data were analyzed according to the comparative ΔΔCt method and normalized by using β-Actin housekeeping gene. Sequence of primers used in this study:

Nestin forward 5′-GGAGAAGGACCAAGAACTG-3′, reverse 5′-ACCTCCTCTGTGGCATTC-3′; β3-tubulin forward 5′-TCAGCGTCTACTACAACGAGGC-3′, reverse 5′-GCCTGAAGAGATGTCCAAAGGC-3′; β-Actin forward 5′-GACAGGATGCAGAAGGAGATTACTG-3′, reverse 5′-CTCAGGAGGAGCAATGATCTTGAT-3′.

### 2.8. Statistical Analysis

Analyses were performed using either a two-sided unpaired Student’s *t*-test or a one-way analysis of variance with a Dunnett’s post hoc test. Experiments were repeated three times in triplicate and values were considered statistically significant for *p* < 0.05 (*), *p* < 0.01 (**), *p* < 0.001 (***), *p* < 0.0001 (****).

## 3. Results and Discussion

### 3.1. Generation of Vertically-Aligned Silicon Micropillar Array Structures

Biomimetic cortical-like 3D platforms have been created based on different approaches, such as cell spheroids, organoids and engineered constructs based on hydrogels [34,35,36]. These structures present advantages based on cell self-assembly or enabling spatial self-organization. Nonetheless fine control over the 3D micro-architecture, phenotype and reproducibility have been reported to be challenging [37]. To overcome these limitations, we sought to develop a novel scaffold-based approach mimicking the structural organization of the developing cerebral cortex by ideally controlling network topography.

Mammalian cerebral cortex is a complex layered structure organized in columns generated during developmental stages by means of cortical progenitors (CPs) and neuroblasts that move radially along radial glia fibers serving as scaffolds for directed columnar migration [25,26]. Our aim here is to fabricate a 3D culture platform that mimics in vitro these cerebral radial structures, being able to optimally support the outer growth (from the bottom to the top) of CPs such as that occurring in vivo during cortical neurogenesis. With this aim we designed micropatterned structures containing topographically ordered micropillar arrays. Micropatterned substrates with different geometries have been reported in the form of discontinuous micro-grooved configurations and discontinuous geometries like nanopillar arrays made from a several materials comprising polymers, such as PS, PLGA and PDMS together with hard materials, such as silicon and quartz [18,38,39,40,41]. In particular, in the neural field pillars and cone geometries at micron scale have been shown to control the outgrowth of neuronal processes, guiding neurite outgrowth alignment and cell growth [20,38,42,43].

**Figure 1:**
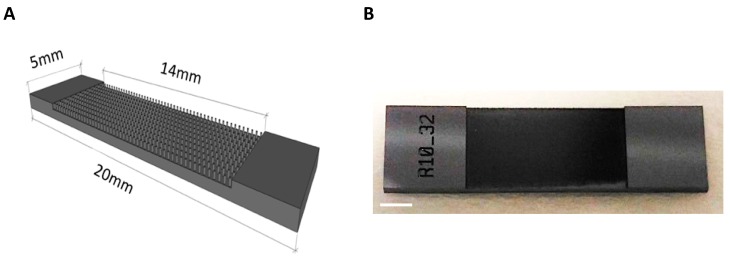
Design, size and morphology of 3D silicon micropillar array slides. (**A**) Schematic structure of a silicon slide containing vertically-aligned micropillars (micropillars are not in scale). (**B**) The image shows a 3D silicon slide. Scale bar: 1 mm.

Here, we produced a novel 3D neural cell culture platform based on silicon substrates displaying arrays of micropillars fabricated by lithographic patterning (see Methods) of crystalline silicon (Si) wafers. Such technique enables the fabrication of discontinuous micropillars exhibiting at the same time anisotropic geometry. The silicon structures consist of a rectangular seeding surface of 0.7 cm^2^ containing vertically arranged micropillars (Figure 1A,B).

Silicon-based nanostructures exhibit conceivable applications in several fields. As examples, plasmonic nanostructures based on array of silicon nanopillars could be used for surface enhanced Raman spectroscopy (SERS) or to control the wettability of a silicon surface [44,45,46,47]. Silicon micropillar arrays can be assembled by lithographic techniques allowing tight control over the size and density of the micropillars, differently from randomly generated rough surfaces as those presented in several other works [17,43,48,49]. Accordingly, they allow greatest control over the topographic structure of the system, thus warranting high reproducibility and robustness of the experiments. Additionally, differently from other materials, silicon surfaces hold particular technological impacts and potential.

The size of the slide was specifically designated to easily accommodate in a well of a tissue culture 12-multiwell plate (Figure S1A,B). By varying the reaction conditions of growth, we can produce pillars that have 10–15 μm diameter and height that can be regulated in the range of 200–600 μm. For this study we used silicon slides with 250 μm tall micropillars. Different micropillars topographies were successfully fabricated by using different masks. We produced 12 distinctive layouts of silicon micropillar arrays, coded as A1 to S6, with variable micropillar density and topographies (square grid-aligned and hexa grid-staggered micropillar arrays and with variable distances between the micropillars; see Table 1).

Additionally, to set up the best conditions for cell growth, we fabricated both oxidized silicon (SiO_2_) and silicon nitride (Si_3_N_4_) vertically-aligned silicon micropillar arrays.

**Figure 2:**
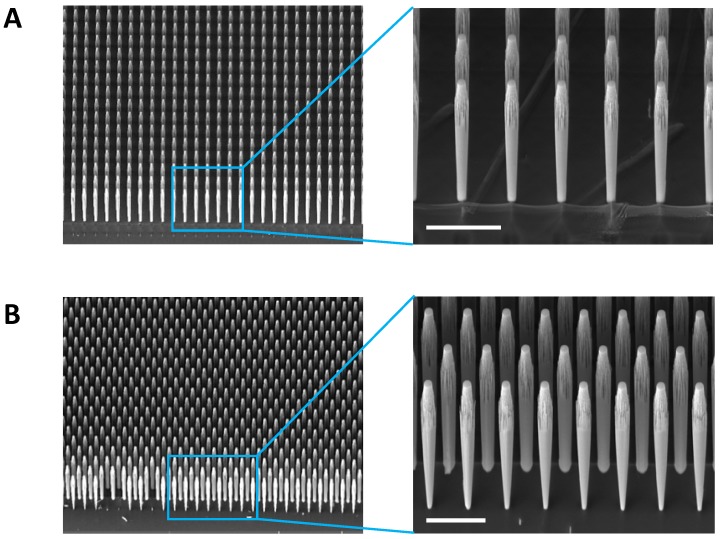
Silicon micropillars can be arranged with different topographies. Silicon micropillar arrays are perfectly vertically-aligned with a height that can be tuned in a 200–600 μm range. Specific spacing, density and morphology of the silicon pillars can be arranged by changing the mask of lithographic process. Low and high magnification of cross-sectional SEM images of aligned (**A**) and staggered (**B**) micropillar arrays. Scale bar: 50 µm.

The fabrication accuracy and morphology of the structures at the nanoscale were evaluated by measuring the distance between the micropillars and their diameter by scanning electron microscopy (SEM). Cross-sectional SEM images were produced to derive the internal structure of the samples (Figure 2A,B) and top-view SEM images to derive the density of the silicon micropillar arrays.

We found that while the distance between micropillars was quite accurate, the diameter of the micropillars varied depending on the height of the structure. Indeed, the diameters at the bottom of the micropillars were reduced of about 40% (~5–7 µm) with respect to the top of the same micropillar.

This effect is particularly noticeable on the first row of micropillars, and we assumed that this is mainly due to the fact that the chemical-physical processes on which the Bosch process is based depending on the area exposed to the engraving process. Therefore, the etching rate is different in the areas between the micropillars than in the areas between the slides. Therefore, we assume that the first row of micropillars is etched much faster than the others and that this is the cause of the reduction of the diameter of the micropillar. Up to now, we were able to reach structures with a diameter of 15 µm and a height of 600 µm, nevertheless further tests to improve this aspect ratio are ongoing.

### 3.2. Culturing Human Cortical Progenitors on Vertically-Aligned Silicon Micropillar Arrays

**Figure 3:**
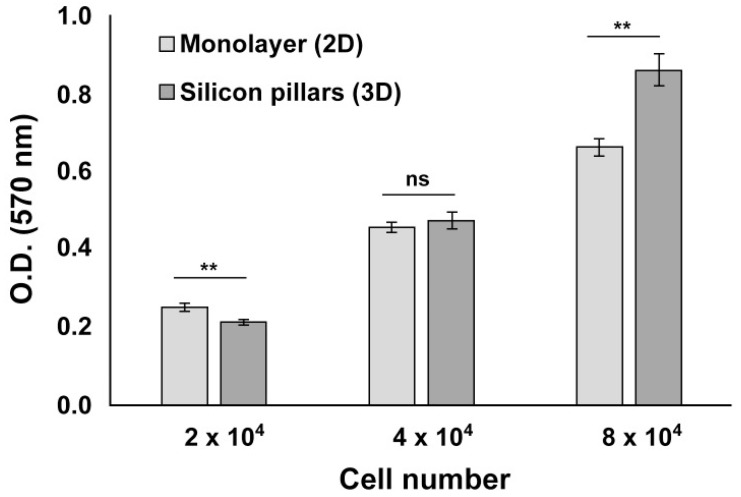
hCPs seeded on silicon micropillar arrays maintain their viability. MTT assay performed on hCPs plated on 3D silicon slide and in standard 2D monolayer shows that hCPs efficiently maintain their viability. Different cell densities were assessed 48 h after seeding. *p* < 0.01 (**), not significant (ns).

To establish which human cortical progenitors (hCPs) density was the most comparable to the standard 2D monolayer method used as control and to detect potential toxic effects of silicon material, we first performed a cell viability assay. hCPs were seeded on laminin-coated samples A6–S6 with cell density ranging from 2 × 10^4^ to 8 × 10^4^ cells per 3D device (i.e., 2.8 × 10^4^ and 1.1 × 10^5^ cells per cm^2^) and cultured for 48 h before being processed in an MTT assay (see Section 2). We found that cell viability was reduced for the lowest cell density (2 × 10^4^ cells) and increased for the highest (8 × 10^4^ cells), compared to the 2D culture. Indeed, 4 × 10^4^ cells per 3D device showed no differences compared to the 2D monolayer culture (Figure 3).

We found no significant difference between oxidized silicon or silicon nitride material (not shown), thus indicating that these 3D structures are fully compatible with living cells without affecting cell growth. Moreover, our silicon micropillar arrays might also allow for increased hCPs proliferation activity in long-term cultures by offering a 3D environment in which the cells can also exploit the third (vertical) dimension for growth. Additionally, we did not detect any significant difference between different topographic layouts and silicon types (oxidized silicon or silicon nitride) tested in terms of cell adhesion, viability and growth (not shown).

**Figure 4:**
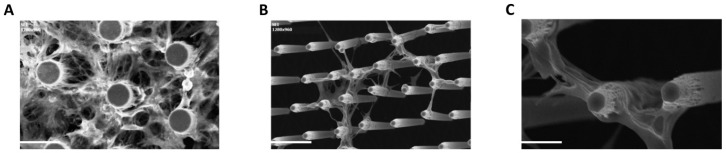
hCPs seeded on silicon slide establish close interactions with micropillars. (**A**) Top-view SEM image showing hCPs seeded at standard density on silicon micropillars. (**B**,**C**) Cross-sectional SEM images showing hCPs seeded at low density on silicon structures to visualize single cells-micropillars interactions. Scale bar: 20 μm (**A**,**B**) and 10 μm (**C**).

As further confirmation of these results, an analogous MTT analysis was performed on mouse radial glia-like NS cells plated at the same aforesaid cell densities (Figure S2A). NS cell analysis gave similar results to the hCPs, indicating that 4 × 10^4^ cells per 3D device represents the preferred cell density. This was then selected as standard seeding density in the subsequent experiments in this work. The compatibility of oxidized silicon or silicon nitride for in vitro functional studies on neurons has been already reported, nevertheless, this is the first study to report compatibility with hCPs [50]. Furthermore, contrary to other studies employing silicon uncoated surfaces, we used surface topographies coated with laminin to optimize cell adhesion as also been reported by others [51,52,53]. In addition, these results demonstrate that our silicon micropillar arrays can be functionalized according to the cells’ needs. We then performed SEM analysis in order to monitor the behaviour of the hCPs seeded on the arrays in terms of interaction with the vertical silicon micropillars. We found that cells at high density interact with each other and with the micropillars establishing a uniform network (Figure 4A).

Interestingly, hCPs plated at low density show an increased propensity to adhere to the micropillars rather than to the bottom flat surface of the device, with cells that can be found at different heights of the micropillars (Figure 4B,C). Cells formed 3D neural networks and suspended bridges throughout the micropillar height and along and between the micropillar walls. A similar formation of suspended neural process bridges has also been reported with microtowers, microfibres and 2PP-DLW fabricated microstructures [54,55,56,57]. In order to visualize the cells seeded on the silicon micropillar arrays with an inverted microscope, the device has to be set upside down, facing the microscope objectives. To facilitate this operation, we fabricated, by 3D-printing technique, a slide holder support to be placed on a glass bottom dish that allows to visualize the slide with a tilt of 45 degrees, so as to have a 3D visual prospect of the cells (Figure S3). Also, a glass bottom dish was used to place the holder (Figure S2B). To visualize live cultures, we generated eGFP^+ve^ hCPs by infection with lentiviral particles allowing constitutive expression of eGFP cDNA (see Methods). eGFP^+ve^ hCPs were used to live monitor the interactions of hCPs with the device and the single silicon micropillars by time-lapse analysis, and to capture the dynamics of cells movements among the micropillars. We found that hCPs move within the chip and interact with each other and with micropillars. In particular, hCPs can move along the whole micropillar from the bottom to the top (Movie S1) in a manner that resembles the in vivo migration along the radial glia during cortical neurogenesis, and extend processes embracing the micropillars (Movie S2). We also noticed the progressive neurite extension surrounding micropillars at the same height, which might represent the way for the cells to preferentially form a layered architecture.

### 3.3. hCPs Seeded on Silicon Micropillar Arrays Proliferate and form 3D Layered Structures

**Figure 5:**
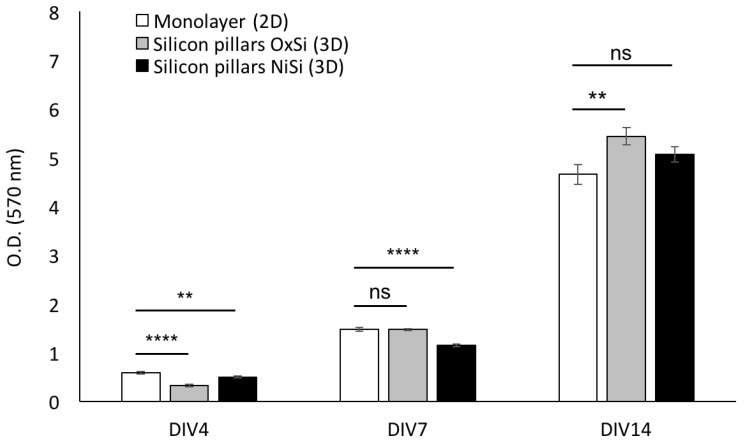
hCPs seeded on silicon slides maintain their proliferation capability. Cell growth analysis (MTT assays) performed on hCPs seeded on oxidized silicon (OxSi) or nitride silicon (NiSi) micropillar arrays. Standard 2D monolayer cultures were used as control. Cultures were assessed at different days in vitro (DIV) after seeding. *p* < 0.01 (**), *p* < 0.0001 (****), not significant (ns).

We next assessed the proliferation of hCPs seeded on different silicon 3D devices in order to find the silicon type better supporting hCPs stable long-term growth. To this aim, hCPs were plated and maintained for several days on silicon oxide (SiO_2_) and on silicon nitride (Si_3_N_4_) micropillar arrays, as well as on plastic dishes as standard monolayer growth conditions. Cultures were analysed by MTT-based growth assays at different time points. Resulting growth curves from the three experimental groups show a modest, yet statistically significant, reduction in cell growth within the first four days for both types of 3D silicon devices. After seven days cultures grown on Si_3_N_4_ devices still exhibit a significant reduction, whereas cultures on SiO_2_ device show no difference with respect to the standard monolayer conditions. At the later time point considered, 14 days, a statistically significant difference was detected for the cells seeded on SiO_2_ 3D silicon device, which resulted in increased growth when compared to the other experimental groups (Figure 5).

**Figure 6:**
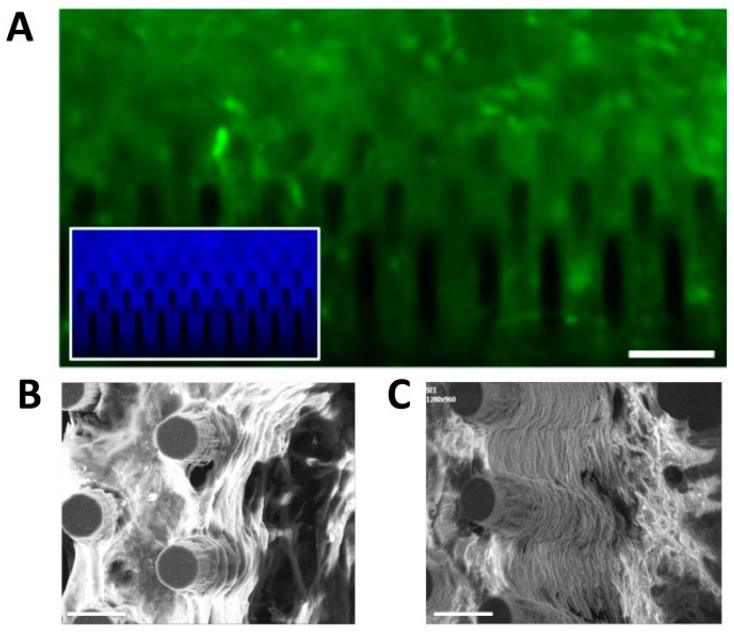
hCPs cultured on silicon micropillar arrays establish layered structures. (**A**) Picture of eGFP^+ve^ hCPs cultured for 14 days on silicon slides showing the establishment of a high-density 3D culture. Inset shows the same culture stained with Hoechst. (**B**,**C**) SEM images of hCPs maintained on silicon pillars device for 14 days. Cultures exhibit the generation of multiple cell layers. Scale bar: 50 μm (**A**) and 20 μm (**B**,**C**).

These results indicate that growth on silicon 3D silicon devices improves hCPs long-term maintenance. Additionally, since oxide silicon 3D arrays resulted to be more compatible and efficient in sustaining hCPs long-term survival and growth with respect with the nitride silicon devices, for the next experiments we employed the former type. Immunofluorescence imaging analysis of hCPs grown on silicon 3D micropillar arrays for two weeks show that cells are well distributed inside the device, filling the whole space among the micropillars (Figure 6A).

Additionally, SEM imaging shows that the cells form a complex three-dimensional multilayered-like structure by growing among the micropillars and interacting with each other, finally establishing regular horizontal layers (Figure 6B,C).

### 3.4. hCPs Grown on Silicon Micropillar Arrays Retain Their Multipotency and Regional Identity

**Figure 7:**
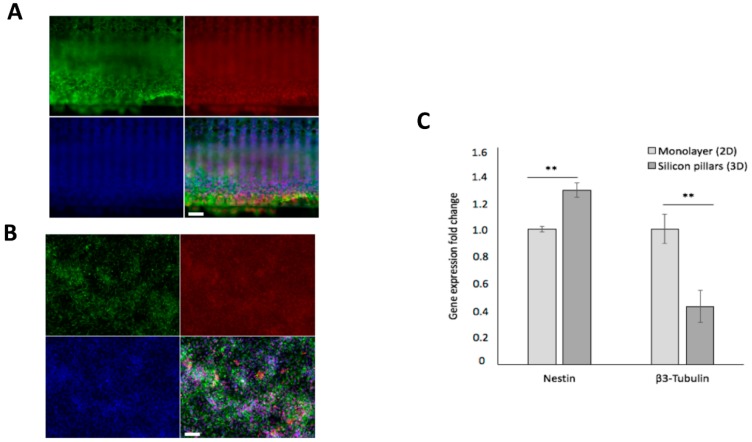
hCPs seeded on silicon slide maintain their neural immature identity. Immunostaining of hCPs for NESTIN (green), SOX2 (red) and nuclear staining with Hoechst (blue) in 3D culture (**A**) and 2D culture (**B**) cultured for 14 days. (**C**) Quantitative RT-PCR assay showing the expression levels of Nestin and β3-Tubulin transcripts in hCPs grown in 2D or 3D cultures. Scale bar: 100 μm (**A**) and 50 μm (**B**). *p* < 0.01 (**), not significant (ns).

We then assessed if growth on silicon 3D arrays might interfere with hCPs (i) multipotency and (ii) preservation of their cortical regional identity. To assess these issues, we first performed an immunostaining analysis for SOX2 and NESTIN, two key neural multipotent markers, on hCP cultures expanded for 14 days on silicon 3D arrays or in standard monolayer conditions. To this respect, we found that silicon 3D cultures show the great majority of cells to co-express SOX2 and NESTIN, comparably to the cultures maintained in standard monolayer conditions previously characterized to efficiently preserve hCPs multipotency (Figure 7A,B and Figure S4).

To extend this result, we measured the transcript levels of Nestin and of the neuronal marker β3-Tubulin by quantitative RT-PCR (Figure 7C). This assay showed a 30% increase and a 60% decrease on the expression levels of Nestin and β3-Tubulin, respectively, in cultures maintained on silicon 3D arrays with respect to standard monolayer conditions (Figure 7C). These results further confirmed that growth on our silicon 3D devices does not alter hCP’s multipotency. Furthermore, growth on silicon 3D arrays further lowers the occurrence of spontaneous neuronal differentiation in the cultures.

**Figure 8:**
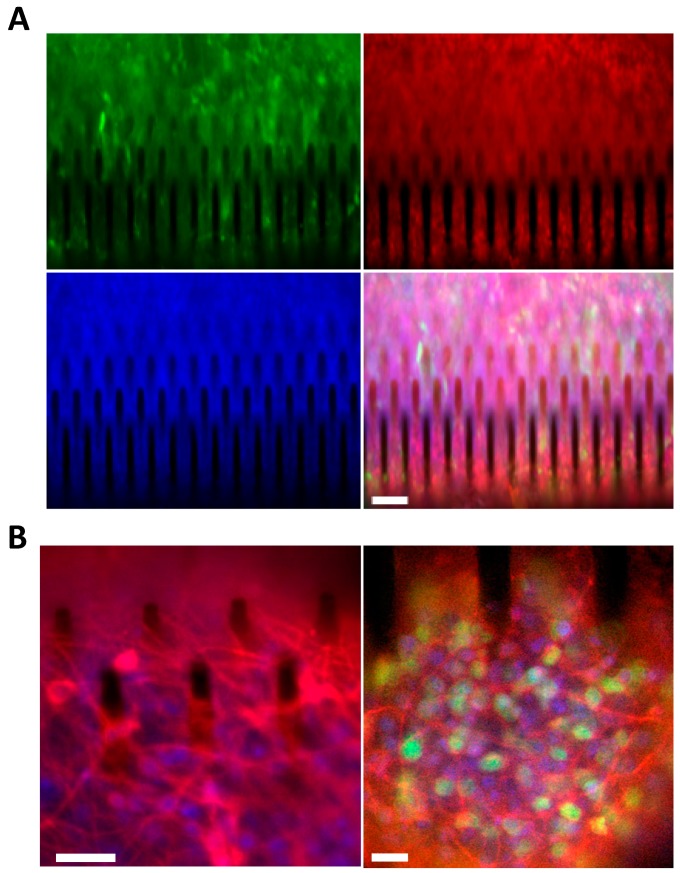
hCPs seeded on silicon micropillar arrays preserve their cortical regional identity and upon exposure to differentiative conditions, generate cortical glutamatergic neurons. (**A**) eGFP^+ve^ hCPs cultured for 14 days on 3D silicon devices preserve the expression of the cortical progenitor marker TBR2 (red). Nuclei are stained with Hoechst (blue). Scale bar: 50 μm. (**B**) hCPs cultured for 5 days on 3D silicon devices and then exposed for 35 days to differentiative conditions maturate into cortical glutamatergic neurons. Left: cultures stained for the pan-neuronal neuronal marker β3-TUBULIN (red). Nuclei are stained with Hoechst (blue). Scale bar: 10 μm. Right: cultures stained for the mature neuronal marker MAP2 (red) and for the cortical neuronal marker CUX1 (green). Nuclei are stained with Hoechst (blue). Scale bar: 10 μm.

We then investigated the capability of hCPs cultured in silicon 3D arrays to retain their original cortical identity. To assess this issue, we first performed an immunostaining analysis for TBR2, a key maker of cortical progenitors, on hCP cultures expanded for 14 days on silicon 3D arrays or in standard monolayer conditions (Figure S5). We found that TBR2 immunoreactivity was maintained in nearly all of the cells in the silicon 3D cultures (Figure 8A; Movie S3). Finally, we tested the ability of hCPs plated in silicon 3D arrays to undergo neuronal maturation giving rise cortical glutamatergic neurons. To this end, hCPs were cultured for 5 days on 3D silicon devices and then exposed for 35 days to differentiative conditions and processed for immunofluorescent analysis for β3-TUBULIN, the mature neuronal marker MAP2 and the cortical neuronal marker CUX1. We found that differentiated cultures exhibited the presence of β3-TUBULIN^+ve^ neuronal cells (Figure 8B, left panel) and the appearance of neurons co-expressing MAP2 and CUX1 (Figure 8B).

These results demonstrate that hCPs grown on silicon micropillar arrays keep their multipotency and cortical identity and are able to generate glutamatergic cortical neurons. However, further experiments are required to further investigate whether the cortical neurons obtained in the 3D environment are able to organize themselves into defined upper and deeper cortical neuronal layers.

## 4. Conclusions

We have described a novel culture platform for hCPs growth based on 3D vertically-aligned silicon micropillar arrays. This structure mimics the radially-oriented cortical radial glia fibres that during embryonic development are essential to control the expansion, radial migration and differentiation of hCPs.

The silicon micropillar arrays can be arranged using different topographic organizations and report micropillar heights not tested with neural progenitors so far. The structures conceived practically combine the advantages of microscale topographies, without the need of complex fabrication techniques. Importantly, our fabrication process allows the production of biocompatible, 3D devices in a highly versatile and reproducible manner. In fact, the micrometre-sized base confers to micropillars good mechanical stability, while establishing a tight interface with the living hCPs.

Other scaffold-based methods have been reported for the biofabrication of in vitro 3D tissue-like models [15,16,17]. In particular, some of these systems, especially the ones based on 3D polymeric materials, i.e., microfibres and hydrogel scaffolds, offer a highly useful and strong method for creating large-scale 3D tissue cultures. Additionally, these systems have been shown to be extremely flexible in terms of production, biocompatibility, biodegradability, mechanical properties and functionalization with chemicals or oligopeptides in order to implement their adhesion and/or ECM mimicking properties [16]. The 3D vertically-aligned silicon micropillar arrays here described exhibited a lower flexibility with respect to these microfibers and hydrogel scaffolds, nevertheless they represent highly reproducible scaffolds in which both the height and the topography of the pillars can be finely regulated. Similar pillar-like structures have been previously reported by Limongi and colleagues and characterized for their ability to allow culturing of functional neuronal and glial cells in a 3D manner, allowing the formation of viable and functional neuronal networks [17]. In particular, the pillars there reported were different from ours since they were designed to reach limited height (cylindrical pillars of 10 μm in height and 10 μm in diameter) and to include a patterning in the nanoscale on their sidewall, leading to a spatial modulation in the z direction. Similarly, culturing platform containing arrays of microchannels arranged into ordered 2D arrays (with maximum height of ~3 μm and amplitudes ranging from ~10 to ~1.5 μm) have been reported [15]. These structures were shown to host neuronal cells strongly guiding their axons’ growth direction and with additional advantage to enable coupling to devices for active sensing and stimulation at the local scale.

Our future efforts should reveal the actual potential of our 3D vertically-aligned silicon micropillar arrays to extensively support hCPs neuronal maturation in order to generate cortical-like tissue comparable to the self-assembled brain organoid technology but with the advantage of an increased reproducibility intrinsic to the scaffold-assisted process.

Additionally, to further exploit the potential of our 3D vertically-aligned silicon micropillar arrays, in the future we aim at coupling this system with a compartmentalized microfluidic device to reach a complete control of culture environment and reduce media volumes and related costs. The acquired knowledge will certainly pave the path towards the generation of valuable tools to study cortical development in humans and for cortical tissue engineering.

## Figures and Tables

**Table 1:** List of different silicon slide arrays fabricated and tested in this work.

Slide Code	Pillar Diameter (μm)	Distance Among Pillars (μm)	Topography
A1	10	20	Alligned
A2	10	30	Alligned
A3	10	40	Alligned
A4	15	20	Alligned
A5	15	30	Alligned
A6	15	40	Alligned
S1	10	20	Staggered
S2	10	30	Staggered
S3	10	40	Staggered
S4	15	20	Staggered
S5	15	30	Staggered
S6	15	40	Staggered

## Data Availability

The raw data required to reproduce these findings are available upon request to the corresponding authors.

## Supplementary Materials

The following are available online at https://www.mdpi.com/2073-4409/9/1/88/s1, Figure S1: Silicon devices were specifically designed to fit into 12 multiwell plates; Figure S2: Analysis of mouse NS cells viability seeded on 3D silicon micropillars arrays; Figure S3: Silicon slide holder device’s picture. The slide lodges on a home-made holder inserted in a bottom glass dish; Figure S4: hCPs seeded on 3D silicon slide preserve their multipotency and create a cell network; Figure S5: hCPs grown in standard 2D conditions exhibit expression of cortical progenitor markers; Video S1: 12.5 hours time-lapse movie performed on eGFP^+ve^ hCPs seeded on silicon micropillars array. A single cell migrating from the bottom to the top of the micropillar is visible; Video S2: 12.5 hours time-lapse movie of eGFP+ve hCPs seeded on 3D silicon slide. Examples of cells extending neurites that interact with pillars are shown; Video S3: Z-stack of eGFP^+ve^ hCPs stained for TBR2 (red) and Hoechst (blue). Cells have been cultured for 14 days on silicon micropillars arrays before the time lapse analysis.
