# Supplementary material for: Vertically-Aligned Functionalized Silicon Micropillars for 3D Culture of Human Pluripotent Stem Cell-Derived Cortical Progenitors
Source: Cells. 2019 Dec 30;9(1):88. doi: 10.3390/cells9010088 (PMC7017050; doi:10.3390/cells9010088)
Supplement: Supplementary file 1 [file cells-09-00088-s001.zip › Cutarelli Supplementary CELLS 22112019/Cutarelli Supplementary figures Cells 2019.pdf]

A

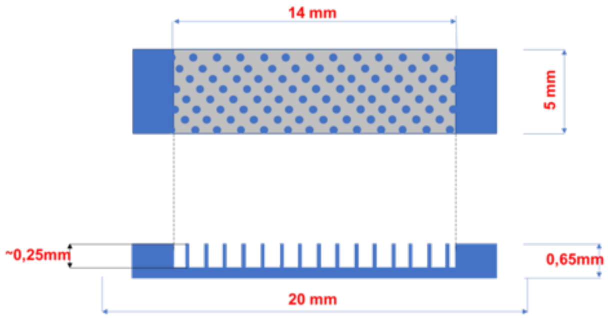

B

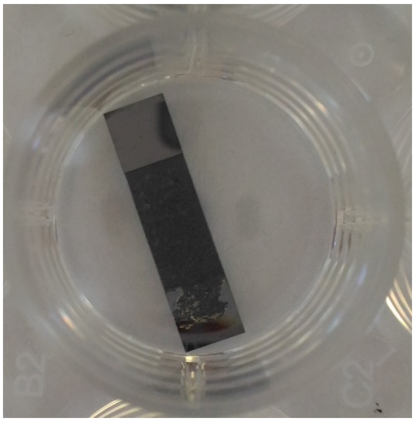

Fig. S1. Silicon devices were specifically designed to fit into 12 multiwell plates. (A) Schematic top-view and lateral-view of silicon devices. (B) Picture of a silicon device lodged into a well of a tissue culture 12-multiwell plate.

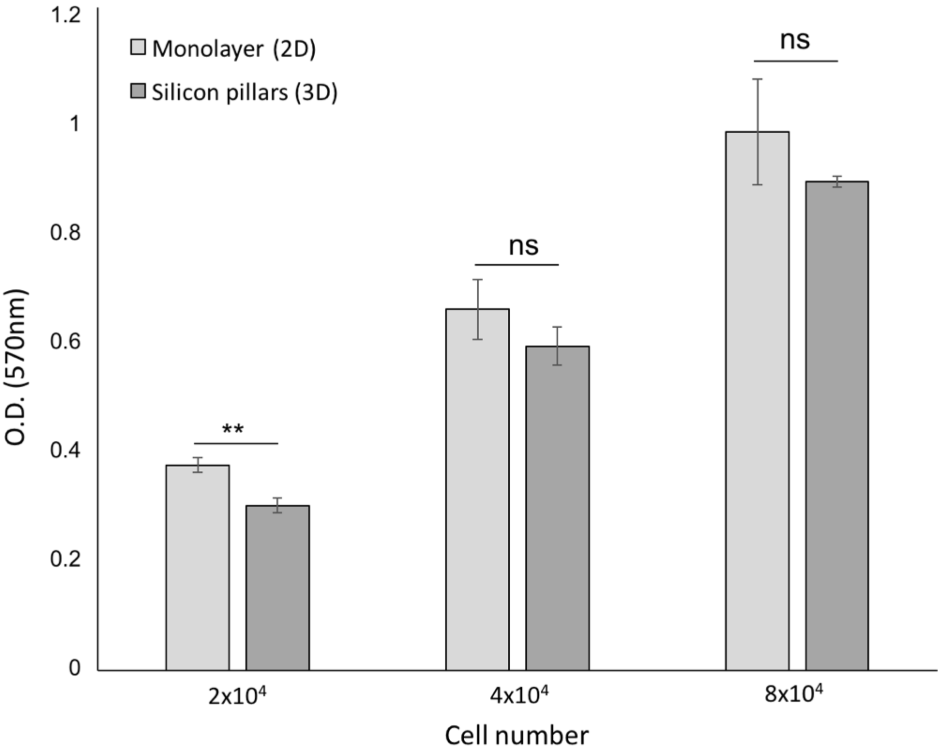

Fig. S2. Analysis of mouse NS cells viability seeded on 3D silicon micropillars arrays. MTT assay performed on cultures plated at different cell densities and grown for 2 days in 2D or 3D conditions.

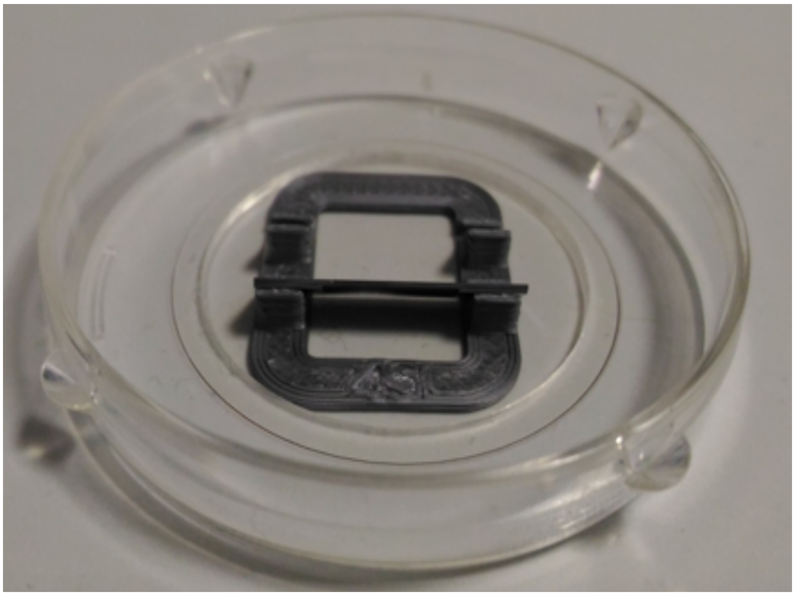

Fig. S3. Silicon slide holder device's picture. The slide lodges on a home-made holder inserted in a bottom glass dish. The holder is conceived to visualize fixed and stained cells on inverted microscopes with a 45° tilting.

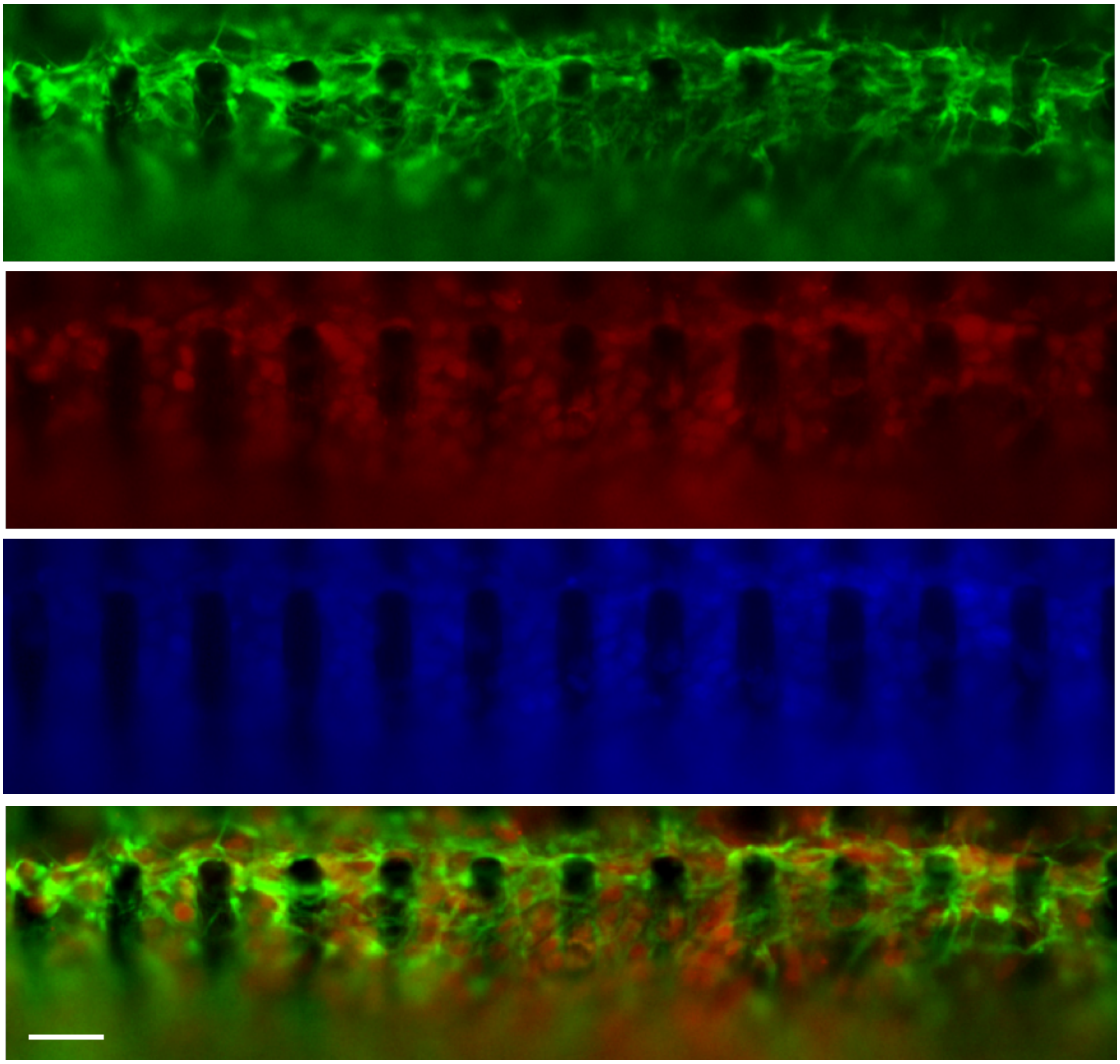

Fig. S4. hCPs seeded on 3D silicon slide preserve their multipotency and create a cell network. hCPs stained for the immature neural markers Nestin (green) and Sox2 (red). Nuclei are stained with Hoescht (blue). Culture exhibits homogeneous distribution and cell-cell and cell-micropillars interactions. Scalebar: 20μm.

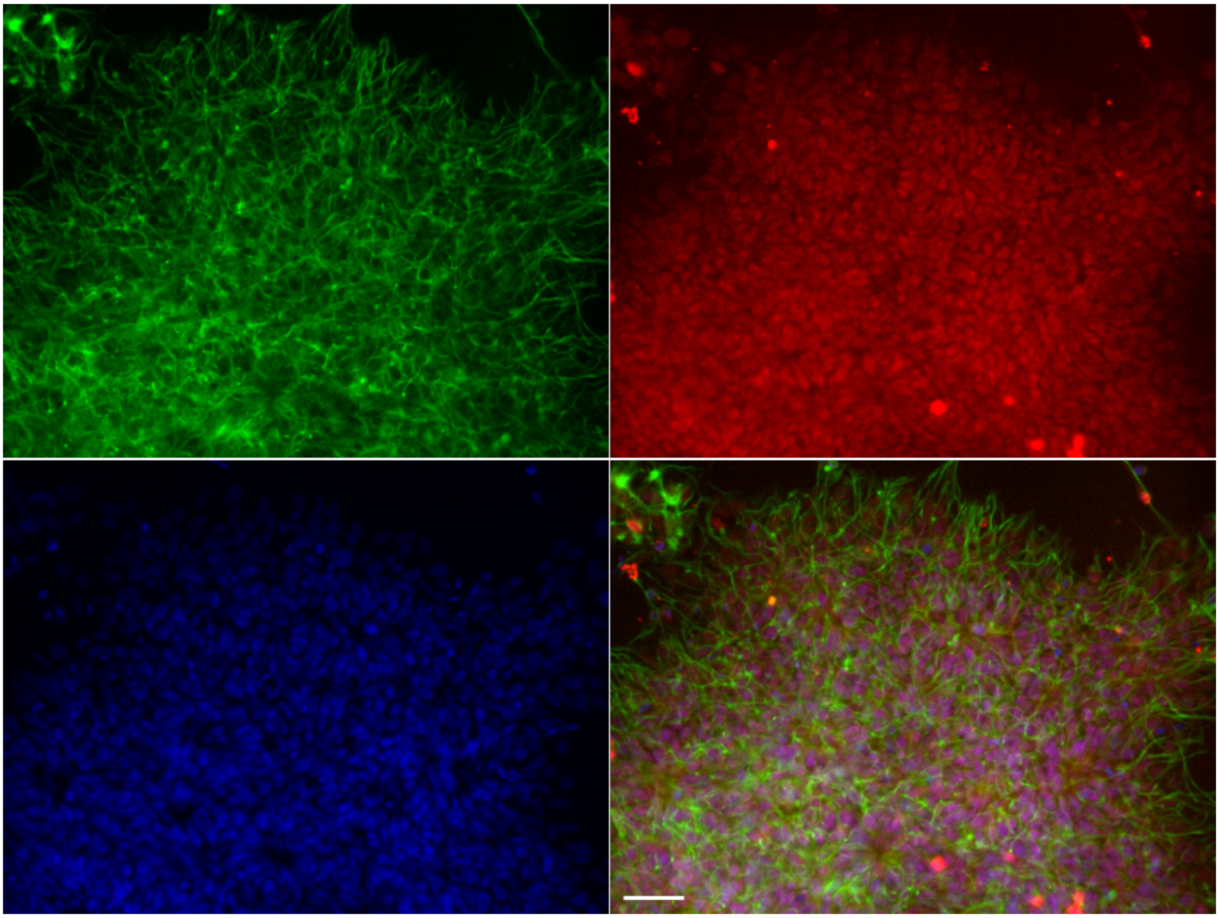

Fig. S5. hCPs grown in standard 2D conditions exhibit expression of cortical progenitor markers. hCPs cultured for 4 days are stained for Nestin (green), TBR2 (red). Hoescht was used for nuclear staining (blue). Scalebar: 100 μm.
